# Effects of two 24-week multimodal exercise programs on reaction time, mobility, and dual-task performance in community-dwelling older adults at risk of falling: a randomized controlled trial

**DOI:** 10.1186/s12889-021-10448-x

**Published:** 2021-11-10

**Authors:** Hugo Rosado, Jorge Bravo, Armando Raimundo, Joana Carvalho, José Marmeleira, Catarina Pereira

**Affiliations:** 1 Departamento de Desporto e Saúde, Escola de Ciências e Tecnologia, Universidade de Évora, Largo dos Colegiais 2, Évora, Portugal; 2 Comprehensive Health Research Center (CHRC), Universidade de Évora, Largo dos Colegiais 2, Évora, Portugal; 3 Faculdade de Desporto, Universidade do Porto, Praça de Gomes Teixeira, Porto, Portugal; 4 CIAFEL - Research Centre in Physical Activity, Health and Leisure, Universidade do Porto, Praça de Gomes Teixeira, Porto, Portugal

**Keywords:** Aging, Falls, Psychomotor intervention, Whole-body vibration, Cognitive function, Physical function

## Abstract

**Background:**

Falls in older adults are considered a major public health problem. Declines in cognitive and physical functions, as measured by parameters including reaction time, mobility, and dual-task performance, have been reported to be important risk factors for falls. The aim of this study was to investigate the effects of two multimodal programs on reaction time, mobility, and dual-task performance in community-dwelling older adults at risk of falling.

**Methods:**

In this randomized controlled trial, fifty-one participants (75.4 ± 5.6 years) were allocated into two experimental groups (EGs) (with sessions 3 times per week for 24 weeks), and a control group: EG1 was enrolled in a psychomotor intervention program, EG2 was enrolled in a combined exercise program (psychomotor intervention program + whole-body vibration program), and the control group maintained their usual daily activities. The participants were assessed at baseline, after the intervention, and after a 12-week no-intervention follow-up period.

**Results:**

The comparisons revealed significant improvements in mobility and dual-task performance after the intervention in EG1, while there were improvements in reaction time, mobility, and dual-task performance in EG2 (*p* ≤ 0.05). The size of the interventions’ clinical effect was medium in EG1 and ranged from medium to large in EG2. The comparisons also showed a reduction in the fall rate in both EGs (EG1: -44.2%; EG2: − 63.0%, *p* ≤ 0.05) from baseline to post-intervention. The interventions’ effects on reaction time, mobility, and dual-task performance were no longer evident after the 12-week no-intervention follow-up period.

**Conclusions:**

The results suggest that multimodal psychomotor programs were well tolerated by community-dwelling older adults and were effective for fall prevention, as well as for the prevention of cognitive and physical functional decline, particularly if the programs are combined with whole-body vibration exercise. The discontinuation of these programs could lead to the fast reversal of the positive outcomes achieved.

**Trial registration:**

ClinicalTrials.gov Identifier: NCT03446352. Date of registration: February 07, 2018.

## Background

By 2050, the number of people aged 60 or more years is expected to double to 2 billion [1]. Additionally, the World Health Organization (WHO) considers aging a determinant risk factor for falls and fall-related injuries [2]. Falls are considered a major public health problem and are associated with injuries, dependence in activities of daily living, disability, and extremely high annual health costs [2, 3].

Falls have a multifactorial etiology based on the relationships between different risk factors [4]. Among the intrinsic risk factors, the deterioration of cognitive and physical functions in older people is particularly evident [4]. The aging process leads to biological and physiological changes in the brain and cognitive function, with effects on reaction time (RT) and dual-task (DT) performance [5, 6]. Importantly, the scientific community has established clear evidence that there are strong associations between cognitive function and the risk of falling; specifically, increases in RT have been consistently shown to be related to falls, and the association is so strong that RT is reported as one of the most important and sensitive indicators of changes in the central nervous system [4, 7]. Lajoie and Gallagher’s study showed that fallers also have a slower RT than do non-fallers [4]. In addition, a reduced ability to perform two tasks simultaneously (e.g. a cognitive task while walking), referred to as DT, has been associated with an increased risk of falls [6]. Concerning physical function, the sensorimotor and neuromuscular impairments that result from aging are associated with reduced levels of mobility and are considered risk factors for falls [8]. Therefore, exercise-based fall prevention programs should modify the complexity and intensity of tasks, particularly those related to mobility and cognitive training, according to the participant’s capacity [8].

Previous studies have shown that single cognitive training programs can induce positive effects on fall risk factors in community-dwelling older adults [9]. These positive effects have also been observed in studies involving single physical training programs. However, several studies on fall intervention programs have shown that exercise alone is one of the most effective interventions to reduce falls in community-dwelling older people [10], but exercise alone may not be enough to improve cognitive functions, especially in terms of DT performance [11]. Despite cognitive or physical training programs being able to induce positive effects on fall risk factors, studies in the literature have shown that multimodal exercise programs have additional advantages [12]. In fact, recent systematic reviews and meta-analysis [5, 13] demonstrated the additional benefits of multimodal exercise programs combining cognitive with physical training for older adults. However, no definitive conclusions have been drawn, showing the need for additional investigations, particularly on the effects of multimodal exercise programs on fall risk factors.

A psychomotor intervention is a therapy that uses the body and movement as intervention mediators to optimize cognitive, motor, and relational competences of psychomotor functioning, through a holistic view [14], and has been shown to prevent the sensorimotor and neurocognitive declines associated with aging [15]. Regarding the whole-body vibration (WBV) intervention, a recent systematic review and meta-analysis suggested that WBV may prevent fractures by reducing falls and improving determinants of falling, particularly physical function-related risk factors [16]. WBV may also improve cognitive function [17]. Nonetheless, as these two methods are reported to potentially be beneficial, it is not known whether an intervention program that combines both methods had additional benefits.

To the best of our knowledge, only one study [18] has implemented a psychomotor intervention program in community-dwelling older adults to reduce the risk for falls. Given the lack of studies about these intervention programs, new and effective interventions that can prevent and reduce falls and thus its consequences, such as fall-related injuries or associated health costs, are needed [7].

Therefore, this study aimed to investigate the effects of two multimodal exercise programs on RT, mobility, and DT performance in community-dwelling older adults at risk of falling.

## Methods

### Trial design

A single-blinded randomized controlled trial (RCT), including a 24-week intervention, 12-week no-intervention follow-up period, and with a parallel three-arm design, was conducted between March 2018 and January 2019. Three groups of community-dwelling older adults from Évora (Portugal) were compared: experimental group 1 (EG1) was enrolled in a psychomotor intervention program, experimental group 2 (EG2) was enrolled in a combined exercise program (psychomotor intervention program + WBV), and the control group (CG) maintained their daily level of physical activity. This study followed the CONSORT guidelines for RCTs (http://www.consort-statement.org). The protocol was registered in ClinicalTrials.gov (NCT03446352), and no significant changes were made.

### Participants

The participants were community-dwelling older adults and were recruited via pamphlets distributed in strategic locations and verbal communication (recreational and senior centers). The minimum sample size needed was estimated to be 15 participants/group, for a total of 45 participants, by the online G*Power software, with α = 0.05 and power = 0.95. The sample size was increased to a minimum of 60 participants (20 in each group) to account for the expected dropout rate of 20%.

The inclusion criteria were: 1) male or female community-dwelling older adults who were aged ≥65 years; 2) had a moderate or high level of physical independence (≥ 18 points), as assessed by the 12-item Composite Physical Function (CPF) scale [19]; and 3) reported at least one fall in the previous 6 months or who were at high risk of falling (a score of ≤25 points on the Fullerton Advanced Balance Scale) [20]. The exclusion criteria were: 1) cognitive impairment, as assessed by the Mini-Mental State Examination (MMSE ≤22 points) [21]; 2) the presence of motor impairment compromising program participation; 3) a musculoskeletal condition (diagnosis of severe osteoporosis [index T ≤ − 2.5], lower limb fracture < 4 months ago, hip or knee prostheses); 4) a cardiovascular condition (e.g. pacemaker); 5) a neurological condition (epilepsy, loss of consciousness leading to a fall [e.g. vertigo syndrome]), tumors or metastases [22]; and 6) participation in a structured exercise program in the previous 6 months [23].

**Fig. 1:**
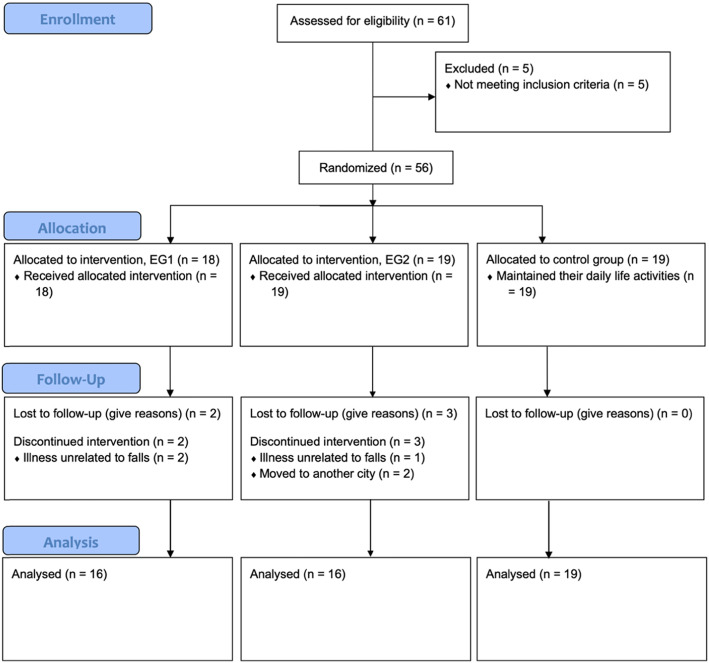
Flow diagram of participant’s recruitment

Sixty-one participants were enrolled in this study (Fig. 1). Five participants were excluded: 2 were excluded due to the presence of motor impairment, and 3 were excluded because they did not report experiencing at least one fall in the previous 6 months or were not at high risk of falling. A total of 56 participants met the inclusion criteria (47 women and 9 men) and were randomly assigned to three groups, with an allocation ratio of 1:1:1, with sequential numbers using the online “random team generator” (https://www.randomlists.com/team-generator). A total of 18 participants were included in EG1, 19 participants were included in EG2, and 19 participants were included in the CG. From baseline to post-intervention, 5 participants (EG1: 2; EG2: 3) dropped out: 3 dropped out due to an illness unrelated to falls, and 2 dropped out because they moved to another city.

This study was approved by the University of Évora Ethics Committee - Health and Well-Being (reference number 16–012) and was performed in accordance with the Declaration of Helsinki. All participants provided written informed consent.

### Procedures

The participants were assessed individually at baseline, at 24 weeks, and at the 12-week follow-up by the same trained evaluator who had an academic degree in rehabilitation sciences. The measures recorded included cognitive and physical functions, fall occurrence, the results of scales/questionnaires, sociodemographic characteristics, and body composition. The questionnaires and cognitive variable assessment were performed in a quiet room. To familiarize the participants with the assessments, the cognitive and physical outcome assessments included verbal instructions provided by the evaluator and a practice trial before the testing trial. For the physical outcomes assessment, the evaluator also demonstrated the task before each testing trial. The data were collected in laboratories at the University of Évora.

### Outcome measures

#### Reaction time

Simple reaction time (SRT) and choice reaction time (CRT) were assessed in the single and DT conditions by the Deary-Liewald reaction time task (DLRT) [24]. In the SRT task, a stimulus (cross) appeared in one box on the monitor, and the participants had to press a key as quickly as they could each time it appeared. In the CRT task, a cross appeared in one of four boxes on the monitor, and the participants had to press the corresponding key as quickly as they could whenever it appeared. The DT conditions involved asking the participants to simultaneously count by twos (starting at the number 0) while performing the SRT and CRT tasks.

The SRT and CRT tasks in the single and DT conditions included 8 practice trials. There were 20 testing trials for the SRT tasks in the single and DT conditions, and there were 40 testing trials for the CRT tasks in the single and DT conditions. For the SRT and CRT tasks, the response time ranged from 150 to 1500 (ms) and 200–1500 (ms), respectively. Both tasks had an inter-stimulus interval ranging from 1000 to 3000 (ms). The median RT (ms) for the four tasks (SRT in the single and DT conditions; CRT in the single and DT conditions) and the number of errors in the CRT tasks (wrong key presses) were recorded for each participant.

#### Timed up and go test

The timed up and go (TUG) test [19] was used to assess functional mobility. The participants were asked to stand up from a chair (height: 46 cm), walk to the 2.44 m mark as quickly and safely as possible, turn approximately 180 degrees and sit down again. The commands were “Ready? Set, go!”, and the period from when the command “Go!” was given to when the participant sat down completely in the chair was recorded with a stopwatch. Two test trials were performed, and the best time (s) was chosen.

#### Cognitive TUG test

DT performance was assessed by the cognitive TUG (CogTUG) test, which was performed 5 min after the TUG test. This task follows the methodology of the 2.44 m TUG test, and the same instructions with the inclusion of the cognitive task instructions were given to the participants. The participants were asked to perform the TUG test while counting backward from a predetermined number. In the practice trial, the participants were asked to start counting backward by one from 150. At 145, the evaluator said “Go!” and the participants stood up from the chair and executed both tasks simultaneously as quickly and safely as possible. After a recovery period, the participants performed one testing trial. For that trial, they were asked to start counting backward by one from 100. At 95, the evaluator said “Go!” and the participants stood up from the chair and executed the DT.

The CogTUG test variables were assessed in accordance with the methodology proposed by Tomas-Carus et al. [25]; the variables included the time spent on the DT task (s), the number of cognitive errors (n), the number of cognitive stops (n), the number of motor stops (n), and the final number (n) (the last number counted before the participant sat down). All CogTUG test trials were recorded by video for further analysis.

#### Falls

The number of falls in the previous 6 months was assessed at baseline and post-intervention. A fall was defined by WHO [2] “*as an event which results in a person coming to rest inadvertently on the ground or floor or other lower level”*, and a questionnaire was used to determine the type and circumstances of each fall (e.g. indoor/outdoor; accidental fall during a usual or an unusual task; consequent injuries).

#### Secondary outcome measures

The Borg Rating of Perceived Exertion (RPE) scale [26] was used to monitor exercise intensity, with scores ranging from 6 points (very, very light) to 20 points (very, very hard). The Caregiver Treatment Satisfaction (CTS) questionnaire [27] through a “face scale” was used to assess the participants’ satisfaction level, with scores ranging from 1 point (extremely dissatisfied) to 5 points (extremely satisfied). Both the RPE scale and the CTS questionnaire were used to observe the participants’ ability to tolerate the multimodal exercise programs. A questionnaire was used to record the participants’ sociodemographic characteristics. The MMSE [21] was used to assess cognitive status. Body mass index (BMI) was calculated by the formula kg/m^2^, and the weight (kg) and height (m) were measured using an electronic scale (Seca 760, Hamburg, Germany) and a stadiometer (Seca 206, Hamburg, Germany), respectively. The 12-item CPF scale [19] was used to assess physical independence across a wide variety of activities of daily living. The scores of the previous scale ranged from 0 (worst) to 24 (best) points, and participants were categorized as having “a high level of function” (24 points), “a moderate level of function” (18–23 points), and “a low level of function” (< 18 points). Last, the International Physical Activity Questionnaire (IPAQ) [28] was used to assess metabolic expenditure (metabolic equivalent of task [MET]-min/week), which was calculated as follows: time (min/day)*frequency*(days/week)*MET intensity (walking or moderate−/vigorous-intensity activities).

### Multimodal exercise programs

The psychomotor intervention program (24 weeks; 75 min/session; 3x/week on alternate days) included exercises simultaneously promoting motor stimulation (e.g. agility, mobility, body awareness) and cognitive stimulation (e.g. problem-solving, cognitive inhibition, or RT training under single and DT conditions). The combined exercise program included the psychomotor intervention program + WBV program (beginning with 72 + 3 min/session and ending with 69 + 6 min/session, respectively; 3x/week on alternate days). Regarding the WBV program (Galileo® Med35), the vibration amplitude (mm) and resting time between series (s) were always 3 and 60, respectively. Throughout the intervention, the exercise time (s) in the WBV program progressively increased from 45 to 60, the series (n) increased from 4 to 6, and the frequency (Hz) increased from 12.6 to 15. Participants performed the WBV program while they stood without shoes and with bent knees. For the intervention, each EG was divided into two classes until 10 participants. There were no differences between the EG1 and EG2 session classes.

Each session was structured to include a beginning ritual (~ 5 m), a warm-up (~ 10 m), the main section (~ 50 m), a cool down (~ 5 m), and a finishing ritual (~ 5 m). At the initial stage, different muscle groups were activated, increasing the neurophysiological parameters. The main section (multimodal exercises) was focused on the specific objectives through sensory, motor, and neurocognitive activities. This section included exercise periods ranging from 10 to 15 min that alternated between exercises focused on motor stimulation, i.e., physical fitness (e.g. moving around cones with a fitball as fast as possible, forward and backward), and exercises focused on cognitive stimulation, i.e., executive functions (e.g. drawing a 3, 8 and a Z on the floor, reciting the days of the week backwards while walking). During the cool down (e.g. stretching or breathing exercises), the physiological parameters returned to normal. At the finishing ritual, the participants signed an attendance sheet and recorded their exercise intensity level on the RPE scale and their satisfaction level on the CTS questionnaire.

The multimodal exercises were intended to be moderate intensity (~ 13 points on the RPE scale) and were conducted by a therapist with a master’s degree in psychomotor therapy. A sport sciences professor at the university supervised the intervention.

After the study, the CG participants were invited to attend a similar fall prevention program.

### Data analysis

The assumptions of normality and homogeneity were tested through the Kolmogorov-Smirnov and Levene tests, respectively. Since most of the sample variables did not follow a normal distribution, non-parametric statistical analyses were conducted. Between-group comparisons were performed using the Kruskal-Wallis test, and within-group comparisons were performed using the Friedman test; both tests were followed by post hoc pairwise comparisons. The Wilcoxon test was performed for within-group comparisons of the number of falls. The means and standard deviations were calculated for all variables.

The delta value (∆: moment_x_ - moment_x-1_) and the respective proportional change delta value (∆%: [(moment_x_ - moment_x-1_)/moment_x-1_] × 100) were computed for all variables: post-intervention vs. baseline, the follow-up vs. post-intervention, and the follow-up vs. baseline.

The effect size (ES) was calculated using Cohen’s method since the data were not normally distributed [29]. Thus, the ES was calculated as r *=* (Z/ √N) for all analyses to determine the magnitude of the treatment effect and thus the interventions’ clinical significance. Cohen’s thresholds were used, and standardized differences of 0.10, 0.30, and 0.50 indicated small, medium, and large effects, respectively [30].

Analyses were performed using the SPSS software package (version 24.0 for Windows, IMB Statistics). A value of *p* ≤ 0.05 was considered statistically significant for all analyses.

A code was assigned to each participant to preserve their anonymity.

## Results

At baseline, the participant’s characteristics, namely, the sociodemographic characteristics, MMSE, BMI, CPF, IPAQ, and fall occurrence, were similar, and no significant differences were observed between groups (*p* ≤ 0.05), as shown in Table 1.

**Table 1:** Participant’s characteristics at baseline

	EG1 Prevalence or Mean ± SD	EG2 Prevalence or Mean ± SD	CG Prevalence or Mean ± SD	***P***-value
Age (years)	74.3 ± 5.4	74.7 ± 5.5	76.8 ± 5.8	0.407
Sex, female (%)	14 (87.5)	15 (93.8)	13 (68.4)	0.124
Educational level (years)	6.0 ± 2.6	6.1 ± 3.4	7.0 ± 5.3	0.997
MMSE (points)	27.7 ± 1.7	28.2 ± 1.7	28.5 ± 1.6	0.332
BMI (kg/m^2^)	29.1 ± 3.0	28.6 ± 4.3	28.1 ± 4.4	0.648
CPF (points)	21.5 ± 2.7	20.8 ± 2.2	21.5 ± 2.8	0.554
IPAQ (MET-min/week)	927.0 ± 557.9	953.4 ± 638.5	740.4 ± 520.9	0.611
Number of falls within the last six months (n)	1.13 ± 0.8	1.19 ± 1.0	1.11 ± 0.3	0.993

*SD* Standard deviation, *EG1* Experimental group attending the psychomotor intervention program (*n* = 16), *EG2* Experimental group attending the combined exercise program: psychomotor intervention program + WBV (n = 16), *GC* control group (*n* = 19), *MMSE* Mini-Mental State Examination, *BMI* Body mass index, *CPF* Composite Physical Function, *IPAQ* International Physical Activity Questionnaire. Significant differences within groups, *p* ≤ 0.05

Fifty-one participants completed the multimodal exercise programs, and the five dropout participants had characteristics to similar those who completed the study. Seventy-five sessions were held, and the adherence rate was similar between the two EGs (EG1: 82.3% vs. EG2: 84.3%). According to the Borg RPE scale scores, both EGs tolerated the interventions well (EG1: 12.9 ± 0.4 points vs. EG2: 13.2 ± 0.3 points). Additionally, the EGs presented similar levels of satisfaction (EG1: 4.98 ± 0.3 points vs. EG2: 4.99 ± 0.1 points).

At baseline, no significant differences were found between groups in the cognitive and physical functional variables or in the number of falls.

The comparisons within groups concerning the RT variables (Table 2) showed significant differences between the baseline and post-intervention evaluation in both EG2 and the CG. After the 24-week intervention, the CG had poorer results and spent more time performing the “CRT” task (∆%: 10.9%, *p* = 0.045), and EG2 showed improvements in “CRT DT” task performance, as the task time decreased (∆%: − 8.3%, *p* = 0.040). The post hoc pairwise comparisons also revealed significant differences between the post-intervention and follow-up evaluations, in the variable “SRT” within EG2, where the performance decreased, as the participants required more time to perform the task (∆%: 14.3%, *p* = 0.013). For EG2, the ESs for the change from baseline to the post-intervention evaluation in the variable “CRT DT” (r = 0.43), and between the post-intervention and follow-up evaluations in the variable “SRT” (r = 0.44) were medium.

**Table 2:** Impact of the multimodal exercise programs on reaction time

		Baseline (A) (Mean ± SD)	Post-intervention (B) (Mean ± SD)	Follow-up (C) (Mean ± SD)	***P***-value	Pairwise Comparison
Reaction Time						
SRT (ms)						
	EG1	480.2 ± 194.8	390.9 ± 77.3	410.5 ± 109.1	0.444	–
	EG2	448.1 ± 159.5	371.6 ± 89.4	424.8 ± 134.5	0.047	–
	CG	418.7 ± 143.6	460.5 ± 192.1	463.6 ± 196.7	0.104	–
SRT DT (ms)						
	EG1	676.3 ± 218.6	569.7 ± 223.2	605.6 ± 208.6	0.099	–
	EG2	621.1 ± 201.8	516.3 ± 149.5	599.5 ± 232.4	0.185	–
	CG	576.9 ± 121.2	600.2 ± 219.3	577.5 ± 169.8	0.854	–
CRT (ms)						
	EG1	935.1 ± 166.1	908.0 ± 154.9	909.9 ± 186.9	0.444	–
	EG2	927.1 ± 179.5	857.4 ± 168.2 ^a^	924.4 ± 155.4	0.144	–
	CG	916.4 ± 172.7	1015.9 ± 177.4	962.8 ± 197.9	0.050	A < B
CRT errors (n)						
	EG1	1.3 ± 1.8	0.7 ± 1.2	0.9 ± 1.1	0.636	–
	EG2	0.5 ± 0.7	0.6 ± 0.9	0.9 ± 1.1	0.172	–
	CG	0.6 ± 0.8	0.3 ± 0.6	0.3 ± 0.7	0.328	–
CRT DT (ms)						
	EG1	1070.4 ± 141.6	996.9 ± 203.8	1012.8 ± 155.2	0.444	–
	EG2	1035.0 ± 164.7	949.5 ± 171.8 ^a^	1054.3 ± 188.9	0.022	A > B
	CG	1036.6 ± 173.0	1092.3 ± 161.3	1064.3 ± 189.6	0.128	–
CRT DT errors (n)						
	EG1	1.1 ± 1.6	0.9 ± 1.3	0.5 ± 1.0	0.307	–
	EG2	0.9 ± 1.2	0.7 ± 1.0	0.6 ± 1.3	0.598	–
	CG	0.9 ± 1.5	0.7 ± 1.2	0.6 ± 1.0	0.770	–

*SD* Standard deviation, *EG1* Experimental group attending the psychomotor intervention program (*n* = 16), *EG2* Experimental group attending the combined exercise program: psychomotor intervention program + WBV (*n* = 16), *CG* Control group (*n* = 19), *SRT* Simple reaction time, *DT* Dual-task, *CRT* Choice reaction time. > or <: significant differences within groups, *p* ≤ 0.05. ^a^: significant differences between *EG2* and *CG*, *p* ≤ 0.05

The comparisons between groups in the RT variables showed significant differences only at the post-intervention evaluation. Those differences were evident between EG2 and the CG, particularly in the variables “CRT” and “CRT DT”. For the “CRT” variable, EG2 performed better than the CG did, as the participants needed less than 158.5 ms to perform the task; for the variable “CRT DT”, EG2 performed better than the CG, as they spent less than 142.8 ms on the task (*p* ≤ 0.05). Concerning the ESs between EG2 and the CG, it was medium in the variables “CRT” (r = 0.46) and “CRT DT” (r = 0.44).

Concerning the mobility and DT performance variables (Table 3), the comparisons within groups revealed significant differences, particularly in the EGs. Improvements were observed in both EGs between the baseline and post-intervention evaluation in the “TUG” mobility variable (EG1∆%: − 7.0%, *p* = 0.011; EG2 ∆%: − 12.2%, *p* = 0.004) and in the “CogTUG” DT variables, namely, in “time” (EG1∆%: − 10.8%, *p* = 0.002) and in “cognitive stops” (EG2∆%: − 90.9%, *p* = 0.006). The post hoc pairwise comparisons also revealed significant improvements in the “cognitive stops” variable within EG1 (∆%: − 66.7%, *p* = 0.020). Additionally, significant differences were observed in both EGs from post-intervention to the follow-up evaluations, where the performance at the follow-up decreased, particularly in the “TUG” mobility variable (EG1∆%: 12.1%, *p* = 0.002; EG2: 15.4%, *p* = 0.024), in the CogTUG variables, namely, “time” (EG1∆%: 11%, *p* = 0.024; EG2∆%: 16.5%, *p* = 0.014) and the number of cognitive errors (EG2∆%: 166.7%, *p* = 0.040). Concerning the CG, differences were observed between the baseline and the follow-up evaluation only in the “TUG” variable, in which the CG required more time to perform the task (∆%: 11.4%, *p* = 0.017). Regarding these variables, the ES of the changes within groups between the baseline and the post-intervention evaluation ranged from 0.41 (medium) to 0.49 (medium) in EG1 and ranged from 0.47 (medium) to 0.53 (large) in EG2, while the ES between post-intervention and follow-up evaluations ranged from 0.41 (medium) to 0.57 (large) in EG1 and from 0.41 (medium) to 0.55 (large) in EG2. These ESs regarding the changes over follow-up period show that the performance decreased markedly.

**Table 3:** Impact of the multimodal exercise programs on agility and dual-task performance

		Baseline (A) (Mean ± SD)	Post-intervention (B) (Mean ± SD)	Follow-up (C) (Mean ± SD)	***P***-value	Pairwise Comparison
Mobility						
TUG (s)						
	EG1	7.1 ± 1.3	6.6 ± 1.0	7.4 ± 1.1	0.001	A > B; B < C
	EG2	7.4 ± 1.6	6.5 ± 1.0	7.5 ± 1.9	0.003	A > B; B < C
	CG	7.0 ± 1.5	7.5 ± 1.8	7.8 ± 2.2	0.021	A < C
CogTUG						
Time (s)						
	EG1	10.2 ± 2.9	9.1 ± 2.3	10.1 ± 2.8	0.001	A > B; B < C
	EG2	10.1 ± 2.5	9.1 ± 2.0	10.6 ± 2.7	0.015	B < C
	CG	9.5 ± 3.1	10.2 ± 3.3	10.7 ± 3.4	0.692	–
Cognitive errors (n)						
	EG1	1.0 ± 1.3	0.8 ± 0.8	1.3 ± 1.1	0.262	–
	EG2	1.1 ± 0.8	0.6 ± 0.9	1.6 ± 1.3	0.012	B < C
	CG	0.8 ± 1.0	0.8 ± 1.3	0.8 ± 1.1	0.682	–
Cognitive stops (n)						
	EG1	0.9 ± 1.0	0.3 ± 0.5	0.5 ± 0.7	0.020	–
	EG2	1.1 ± 0.8	0.1 ± 0.3	0.6 ± 0.6	< 0.001	A > B
	CG	0.8 ± 0.7	0.6 ± 1.0	0.5 ± 0.8	0.148	–
Motor Stops (n)						
	EG1	0.3 ± 0.4	0.2 ± 0.4	0.3 ± 0.4	0.819	–
	EG2	0.3 ± 0.5	0.1 ± 0.3	0.1 ± 0.3	0.074	–
	CG	0.3 ± 0.6	0.2 ± 0.5	0.3 ± 0.5	0.651	–
Final number (n)						
	EG1	88.9 ± 2.4	88.6 ± 2.9	88.4 ± 2.8	0.328	–
	EG2	88.6 ± 2.3	88.0 ± 2.8	88.1 ± 3.5	0.346	–
	CG	88.6 ± 2.8	88.4 ± 2.7	88.1 ± 2.7	0.302	–

*SD* Standard deviation, *EG1* Experimental group attending the psychomotor intervention program (n = 16), *EG2* Experimental group attending the combined exercise program: psychomotor intervention program + WBV (n = 16), *CG* Control group (n = 19), *TUG* timed up and go, *CogTUG* Cognitive timed up and go test. > or <: significant differences within groups, *p* ≤ 0.05

No significant differences between groups were observed in the mobility and CogTUG variables (*p* ≤ 0.05).

Regarding the number of falls, the comparisons within groups between the baseline and the post-intervention showed that the number of falls decreased in both EGs (EG1: 1.13 ± 0.8 vs. 0.63 ± 0.7, *p* = 0.021; EG2: 1.19 ± 1.0 vs. 0.44 ± 0.7, *p* = 0.007), while that in the CG remained the same (1.11 ± 0.3 vs. 0.95 ± 1.0, *p* = 0.405). No significant differences between groups were observed (*p* ≤ 0.05).

## Discussion

This RCT showed that both multimodal exercise programs designed for community-dwelling older adults at risk of falling were well tolerated and effective for fall prevention. Both intervention programs promoted a decrease in the fall rate and induced clinically significant effects on physical and cognitive risk factors for falls, particularly RT, mobility, and DT performance. The results showed that the magnitude of the treatment effect was higher for the intervention combining the psychomotor intervention program and the WBV exercise program, providing evidence that the intervention program combining both methods has additional benefits. In addition, contrary to other researchers’ findings [31, 32], the follow-up results in the present study showed that the benefits observed in RT, mobility, and DT performance by both intervention programs in community-dwelling older adults were reversed after the programs were discontinued.

The fact that the multimodal exercise programs in this study were supervised, instead of, for example, home-based, may have led to the programs being more effective [33]. Moreover, the adherence rate in the EGs in the present study (83.3%) was slightly higher than that in other studies on 24-week intervention programs (70%) [34] carried out in community-dwelling older adults. Concerning the Borg RPE scale results, the two EGs in the present study showed results similar to those in other studies on moderate-intensity intervention programs in community-dwelling adults [35].

Regarding cognitive function, the within-group comparisons showed that only the combined exercise program induced improvements in the RT variables, particularly in “CRT DT”, with medium ES. These improvements were also evidenced by between-group comparisons, concerning the combined exercise program and the CG, in the variables “CRT” and “CRT DT”. A previous 16-week study by Linde and Alfermann [36] showed that a combined intervention (physical + cognitive) also increases cognitive speed, with a medium ES. However, that 16-week study showed no changes in RT variables in the EG. Few studies in community-dwelling adults have included DLRT evaluations, especially for multimodal exercise programs [37], making the findings of the present study relevant. In the present study, the CG participants demonstrated decreased performance in RT variables, particularly “CRT”, which is in line with the neurocognitive losses associated with aging reported in other studies [5, 6]. Comparing the EGs, although both programs led to improvements in cognitive function, the combined exercise program may have improved RT performance more. To our knowledge, no studies on the effects of active WBV on cognitive function in community-dwelling older adults have been conducted. Few studies have investigated these effects in healthy young adults [17], and they found acute positive effects on cognitive function, despite the study participants having high executive function. At the follow-up evaluation, the benefits from the intervention in EG2 were no longer evident, particularly in the “CRT DT” variable, since no significant differences were found between the baseline and the follow-up evaluations, and in the variable “SRT”. The magnitude of the treatment effect of the combined exercise program in the variable “CRT DT” after the no-intervention follow-up period followed this performance decrease, with a reversed magnitude of 0.44. Consistent with our findings, in a 12-week follow-up study, Linde and Alfermann [36] also found that the ES of the combined intervention decreased from medium to small, and in cognitive speed in particular, the ES decreased from large to medium.

Regarding physical function, the within-group comparisons between the baseline and post-intervention evaluations showed that both multimodal exercise programs induced significant improvements in mobility and CogTUG variables, with a medium ES in EG1, and ranging from medium to large in EG2. Regarding mobility, these results are consistent with those of the study by Freiberger et al. [18], in which the fitness intervention group, focusing more on strength and endurance training, exhibited slightly better TUG test results than did the psychomotor intervention group. The multimodal exercise program studied by Vaughan et al. [37], which was focused on physical function, led to a larger ES in TUG performance than did the programs implemented in the present study; the test time decreased from 6.6 ± 1.4 to 4.9 ± 0.7 s. The slight discrepancy in results between that study and the present study may be related to the fact that the mean age of the EG in the previous study was approximately 5 years younger than those of the two EGs in the present study. The WBV can also lead to improvements in mobility as reported by an 8-week singular WBV intervention study conducted by Yang et al. [38] in community-dwelling adults, measured by the TUG test (9.96 ± 2.49 vs. 9.06 ± 1.60). Furthermore, the comparisons between groups demonstrated that both EGs had similar results concerning mobility, with a medium ES. At the follow-up evaluation, the TUG time increased in all groups (including the EGs). Contrary to EGs in other studies with no-intervention follow-up periods of at least 12 months [31], the EGs in the present study did not exhibit long-term effects of the psychomotor or combined intervention programs regarding mobility, and the TUG time increased by 12.1% in EG1 and by 15.4% in EG2 from post-intervention to the follow-up evaluation. Moreover, the decreasing trend observed in the intervention period continued in the CG, and the TUG time increased by 4.0% during the follow-up period.

Concerning DT performance, both multimodal exercise programs significantly improved the CogTUG variables. However, the combined exercise program induced improvements with larger treatment effects than did the singular psychomotor intervention program. This observation was especially evident in the variable “cognitive stops”, for which the ES of the combined exercise program was 0.53 and that of the psychomotor intervention program was 0.41. This finding is important, as Tomas-Carus et al. [25] suggested that the CogTUG test with the counting numbers backward test may be more effective than the TUG test alone in classifying fallers and non-fallers among community-dwelling older adults, with particular relevance to the cognitive stop and cognitive error results. Thus, the findings of the present study should be considered in the development of fall prevention programs, and these programs should include DT paradigms. The DT results in the present study are in line with those of a 24-week study conducted by Eggenberger et al. [32], which comprised two multimodal exercise programs that included different types of physical exercise and simultaneous cognitive training tasks; the authors observed that these programs significantly improved DT variables to a greater extent than did single interventions involving walking. The findings in the present study are also consistent with those of a 12-week study conducted by Yokoyama et al. [39], which showed that a cognitive-motor DT intervention program induced more benefits than did a single intervention in terms of cognitive domains. The larger treatment effect in the variable “cognitive stops”, within EG2, may be explained by WBV providing additional benefits in the multimodal exercise program. In fact, the sensorimotor and neuromuscular stimulation, promoted by WBV along with the neurophysiological changes induced in DT training, can lead to improvements in cognitive function [6]. At the 12-week follow-up evaluation, in this study, the DT effects were no longer evident, with some variables showing significant declines, particularly the DT performance time and the number of cognitive errors; these findings are contrary to those in the study by Eggenberger et al. [32], in which the improvements in DT performance remained at the 1-year follow-up.

Regarding the outcome “number of falls”, both programs induced changes in the fall rate by decreasing the number of falls (EG1: -44.2%; EG2: − 63.0%). No studies were found that evaluated the effect of a psychomotor intervention program in the fall rate. The 16-week study implemented by Freiberger et al. [18], which included a psychomotor intervention focusing mainly on body awareness and coordination, showed improved physical function performance at the post-intervention, but no reduction in the number of falls at the 12-month follow-up. Although a previous meta-analysis [16] observed that WBV training induced a reduction in the fall rate of 0.67 (95% CI 0.50 to 0.89, *p* = 0.0006), most of the studies included were performed in nursing homes, and compared with the present study, these studies included programs of different lengths or used higher frequencies (≥ 20 Hz). However, although lower vibration frequencies were applied in the present study, beneficial results were obtained without endangering the integrity of the skeletal muscle structures and joints, which can be affected by a higher vibration frequency [40].

Future studies should further investigate the contribution of the WBV to cognitive function and its neurophysiological mechanisms in community-dwelling older adults. A strength of the present study is that it had methodological quality, given that the study design was an RCT and a long-term intervention was implemented; moreover, previously, these two intervention programs were barely studied. However, the present study also has some limitations. First, this study has a single-blinded rather than a double-blinded design. The small sample size and associated dropout rate may have limited the statistical power of the study and thus the ability to generalize the present findings. Nonetheless, the sample size met the minimum size calculated by G*Power in the power analysis, 15 participants per group, and other studies with the same frequency/week and length of the intervention presented identical dropout rates [34]. In the future, the number of falls at the follow-up evaluation should be recorded. Last, 82.4% of the participants in this study were women. Although this proportion is similar to those in other prevention fall programs, recruitment strategies must be adopted to reduce this inequality in sex [41].

## Conclusions

This RCT study showed that the two multimodal exercise programs studied were well tolerated and were effective in improving cognitive and physical risk factors for falls, particularly RT, mobility, and DT performance. Moreover, the improvements induced in these risk factors were concomitant with a significant reduction in the number of falls in both EGs. Both multimodal exercise programs induced positive effects in mobility and DT performance (and in RT in EG2), with a medium clinical effect in EG1 and ranging from medium to large in EG2. These effects were no longer evident after the 12-week no-intervention follow-up period. Considering that falls are a major public health problem, these findings reveal the benefits of the two multimodal interventions in fall prevention programs. Moreover, this study demonstrated the importance of not discontinuing psychomotor intervention programs to prevent the deterioration of cognitive and physical function in community-dwelling older people at risk of falling, particularly when they are combined with WBV exercise.

## Data Availability

The datasets used and/or analyzed during the current study are available from the corresponding author upon reasonable request.

## Abbreviations

WHO: World Health Organization
RT: Reaction time
DT: Dual-task
WBV: Whole-body vibration
RCT: Randomized controlled trial
EG1: Experimental group 1
EG2: Experimental group 2
CG: Control group
CPF: Composite Physical Function
MMSE: Mini-Mental State Examination
SRT: Simple reaction time
CRT: Choice reaction time
DLRT: Deary-Liewald reaction time task
TUG: Timed up and go
CogTUG: Cognitive timed up and go
RPE: Borg Rating of Perceived Exertion
CTS: Caregiver Treatment Satisfaction
BMI: Body mass index
IPAQ: International Physical Activity Questionnaire
MET: Metabolic equivalent of task
ES: Effect size
SPSS: Statistical Package for the Social Sciences
